# Effects of sprint interval training with voluntary hypoventilation on aerobic and anaerobic energy metabolism in young men

**DOI:** 10.1007/s00421-026-06124-w

**Published:** 2026-01-19

**Authors:** Kosei Chiba, Toshihiro Yasuda

**Affiliations:** Faculty of Human Development and Culture, Fukushima University, 1 Kanayagawa, Fukushima, 960-1296 Japan

**Keywords:** Voluntary hypoventilation, Sprint training, VO₂max, Oxygen pulse, Repeated sprint ability, Maximal accumulated oxygen deficit

## Abstract

**Purpose:**

This study aimed to investigate the effects of sprint interval training with voluntary hypoventilation (VHL) on aerobic and anaerobic energy metabolism in young men.

**Methods:**

Fourteen healthy male university students were randomly assigned to a normal breathing group (CON, n = 7) or a VHL group (n = 7). Both groups performed repeated sprint training for three weeks. Pre- and Post-training, we assessed maximal oxygen uptake (VO₂max) as an index of aerobic capacity, and maximal accumulated oxygen deficit (MAOD) and repeated sprint ability (RSA) as indices of anaerobic energy metabolism, together with VO₂, oxygen pulse, and arterial oxygen saturation (SpO₂) during the RSA test.

**Results:**

During training, SpO₂ transiently decreased in the VHL group. After the three-week intervention, VO₂max increased significantly in the VHL group, whereas no significant change was observed in the CON group. During the RSA test, oxygen pulse and VO₂ during the later sprint repetitions were also higher post-training in the VHL group only, while MAOD did not change in either group.

**Conclusions:**

Sprint interval training with VHL enhanced oxidative metabolism through improvements in VO₂max and oxygen pulse, thereby supporting sustained performance during repeated sprints. However, anaerobic metabolic capacity (MAOD) did not improve within the short training period. As VHL requires no altitude exposure or artificial hypoxic devices, it may serve as a practical and accessible training strategy.

## Introduction

Regular physical activity confers extensive physiological benefits, including improvements in exercise performance, physical fitness, and prevention of chronic diseases such as diabetes, hypertension, and cardiovascular disorders (Booth et al. 2012; Warburton, 2006). Despite these well-established benefits, many adults fail to achieve the recommended levels of exercise, and physical inactivity has become a global health concern (Hallal et al. 2012). Lack of time is one of the most frequently cited barriers to regular exercise.

In response to this challenge, high-intensity interval training or repeated sprint training (RST) have attracted increasing attention, as they can induce physiological adaptations comparable to or greater than those elicited by moderate-intensity continuous training, despite a lower total exercise volume. These exercises typically consist of short bouts of near-maximal or all-out efforts (≥ 90% of VO₂max or > 75% of maximal power). Each bout is interspersed with periods of rest or low-intensity activity. Accumulating evidence has shown that RST improves aerobic endurance, anaerobic performance, and symptoms related to metabolic disorders (Cassidy et al. 2017; Weston et al. 2014).

To further enhance training efficacy, altitude training has long been used to improve aerobic capacity through cardiovascular and skeletal muscle adaptations (Billaut et al. 2012). However, altitude training is not always feasible due to economic and geographical constraints. Consequently, artificial hypoxic environments such as hypoxic chambers and hypoxicator systems have been developed. Repeated sprint training in hypoxia (RSH) has been reported as an effective strategy to stimulate both aerobic and anaerobic energy metabolism within a relatively short training period (Faiss et al. 2013). However, the requirement for specialized equipment and facilities limits the accessibility of RSH in many practical settings.

As a simpler alternative to impose hypoxic stress without equipment, voluntary hypoventilation at low lung volume (VHL) has been proposed. This method, involving breath-holding at low lung volume, induces transient hypoxemia through respiratory control. Recent studies have demonstrated the physiological effects of VHL. Woorons et al. (2007) showed that sprinting with breath-holding reduces arterial oxygen saturation, providing a stronger hypoxic stimulus compared with normal breathing. Woorons et al. (2016) further reported that interval training with VHL may enhance peripheral oxygen utilization. Similarly, a review of RSH indicated that improvements in metabolite clearance and upregulation of oxidative enzymes contribute to enhanced endurance performance (Girard et al. 2017). At the molecular level, RSH has been shown to upregulate HIF-1α, VEGF, and PGC-1α, thereby promoting angiogenesis and mitochondrial biogenesis (Faiss et al. 2013). These findings suggest that similar adaptations might occur with VHL. Supporting this notion, Woorons et al. (2017) found that repeated VHL improved blood lactate clearance, buffering capacity, and post-exercise recovery, indicating potential anaerobic adaptations.

Overall, previous research suggests that VHL can induce both aerobic and anaerobic adaptations comparable to RSH, while offering a practical, equipment-free training method. However, experimental evidence remains limited, particularly regarding short-term interventions and performance outcomes. Moreover, most prior studies have focused on aerobic indices such as maximal oxygen uptake (VO₂max), whereas anaerobic metabolism has been insufficiently examined. Blood lactate concentration has often been used as an indirect index of anaerobic capacity, but because lactate concentration depends on the balance between production and clearance, it does not necessarily reflect anaerobic capacity itself. For example, individuals with higher aerobic capacity exhibit faster lactate clearance (Menzies et al. 2010). Therefore, maximal accumulated oxygen deficit (MAOD) provides a more reliable measure of anaerobic energy metabolism.

Given the limitations of hypoxic training, VHL has emerged as a practical method to impose transient hypoxemia without external equipment, thereby offering a feasible alternative for inducing hypoxia-related adaptations. Therefore, this study aimed to investigate the effects of sprint interval training combined with VHL on both aerobic (VO₂max and oxygen pulse) and anaerobic [MAOD and repeated sprint ability (RSA)] energy metabolism in young men. In particular, we examined whether VHL could elicit adaptations comparable to RSH without the need for altitude training or specialized hypoxic equipment.

## Methods

### Participants

Fourteen healthy male university students were recruited for this study (age: 21.4 ± 0.5 years, height: 173.3 ± 5.5 cm, body mass: 66.4 ± 4.5 kg). None of the participants had participated in any structured aerobic or sprint training programs during the previous six months, and none had resided at altitudes above 500 m. The study protocol was approved by the Ethics Committee of Fukushima University (approval number: 2024–40) and conducted in accordance with the Declaration of Helsinki. All participants received verbal and written explanations of the study purpose, experimental procedures, protection of personal information, and potential risks. Written informed consent was obtained prior to participation. Participants were also informed that they could withdraw from the study at any time without disadvantage.

### Study design

Fourteen participants were randomly assigned to either the control group (CON, n = 7) or the voluntary hypoventilation group (VHL, n = 7). The experimental period consisted of five weeks. During the first week, baseline measurements were conducted, which comprised a graded exercise test, maximal oxygen uptake (VO₂max), maximal accumulated oxygen deficit (MAOD), and repeated sprint ability (RSA). According to previous findings showing that six training sessions over three weeks (two sessions per week) improved RSA (Faiss et al. 2013), the present study employed the same training frequency and duration. Accordingly, participants performed six sessions of repeated sprint training over three weeks. In the final week, the same measurements were repeated for Pre- and Post-intervention comparisons.

### Aerobic indices (VO₂max and oxygen pulse)

#### Multistage incremental exercise test and maximal oxygen uptake

A cycle ergometer (Model 232C, COMBI, Japan) was used for a multistage incremental exercise test. After one minute of seated rest on the ergometer, participants performed a 1-min warm-up at 50 W, followed by 3-min stages at 75, 100, and 125 W. From the relationship between oxygen uptake and workload, the individual exercise intensities corresponding to 50% VO₂max and 140% VO₂max were calculated. Oxygen uptake was measured breath-by-breath using a metabolic cart (AE-310S, Minato Medical Science, Japan).

Maximal oxygen uptake was assessed using an incremental protocol in which the workload increased by 10 W every 30 s until volitional exhaustion. VO₂max was determined when at least two of the following criteria were met: a plateau in VO₂ despite increasing workload, a respiratory exchange ratio ≥ 1.2, or attainment of the age-predicted maximal heart rate. Pedaling cadence was maintained at 60 rpm, guided by a metronome.

### Oxygen pulse

Oxygen pulse, defined as the ratio of VO₂ to heart rate and expressed as oxygen uptake per beat, was calculated by dividing VO₂ (ml/min) by heart rate. Heart rate was continuously recorded using chest-lead electrocardiography (Aero Cardiner ML-1200, Fukuda Denshi, Japan), and the mean R–R interval during the measurement period was used for the calculation.

### Anaerobic indices (RSA test and MAOD)

#### Repeated sprint ability (RSA) test

A cycle ergometer (Powermax-VIII, Konami, Japan) was used for the RSA test. After a 5-min warm-up at 50 W, participants performed ten 6-s maximal sprints against a resistance corresponding to 7.5% of their body mass. Each sprint was separated by a 30-s recovery period. During the test, VO₂, heart rate, arterial oxygen saturation (SpO₂), mean power output (MPO), and peak power output (PPO) were continuously measured.

#### Maximal accumulated oxygen deficit (MAOD)

MAOD was determined using the method of Medbø et al. (1988). Participants first rested in a seated position on the ergometer for 5 min, followed by a 10-min warm-up at an intensity corresponding to 50% VO₂max, calculated from the multistage incremental exercise test. Thereafter, participants exercised at ~ 140% VO₂max until exhaustion (approximately 2–3 min). Exhaustion was defined as failure to maintain the target cadence of 90 rpm, indicated by cadence dropping below 85 rpm. After exercise cessation, participants remained seated at rest while VO₂ was continuously measured for 15 min. MAOD was calculated as the difference between the predicted oxygen demand of supramaximal exercise and the actual oxygen uptake, obtained by subtracting resting VO₂ from the accumulated VO₂ during the recovery period.

#### Repeated sprint training

A cycle ergometer (Powermax, Konami, Japan) was also used for the training sessions. After a 5-min light-intensity warm-up, participants performed six 6-s all-out sprints against a resistance corresponding to 7.5% of body mass, completing two sets in total. Each sprint was separated by a 30-s recovery period.

Participants in the CON group performed the training with normal breathing, whereas those in the VHL group were instructed to hold their breath during each sprint. Compliance with breath-holding was visually monitored by the investigator and further verified by measuring arterial oxygen saturation (SpO₂) with a fingertip pulse oximeter (Pulsox-Neo, Konica Minolta, Japan). During the first week of training, post-exercise SpO₂ was significantly lower in the VHL group (87.9 ± 3.7%) compared with the CON group (91.6 ± 2.1%) (p < 0.05).

Participants in the VHL group were instructed to hold their breath throughout each 6-s sprint and to resume normal breathing immediately afterward. To standardize the maneuver, all participants performed 1–2 supervised practice trials before the first training session. SpO₂ was measured immediately after each sprint using a fingertip pulse oximeter, and the lowest value recorded during each session was used for analysis.

### Statistical analysis

All data are presented as mean ± standard deviation (SD). The assumption of normality was assessed using the Shapiro–Wilk test. If the assumption was violated, the non-parametric Mann–Whitney U test was applied. Within-group Pre- and Post-training comparisons were analyzed using the Wilcoxon signed-rank test. Two-way ANOVA with factors group (VHL vs. CON) and time (pre vs. post) was conducted to examine main effects and interactions. Effect sizes (η^2^) were also calculated. A p value < 0.05 was considered statistically significant. Statistical analyses were performed using JASP software (Version 0.19.3).

## Results

### Repeated sprint ability (RSA)

**Fig. 1:**
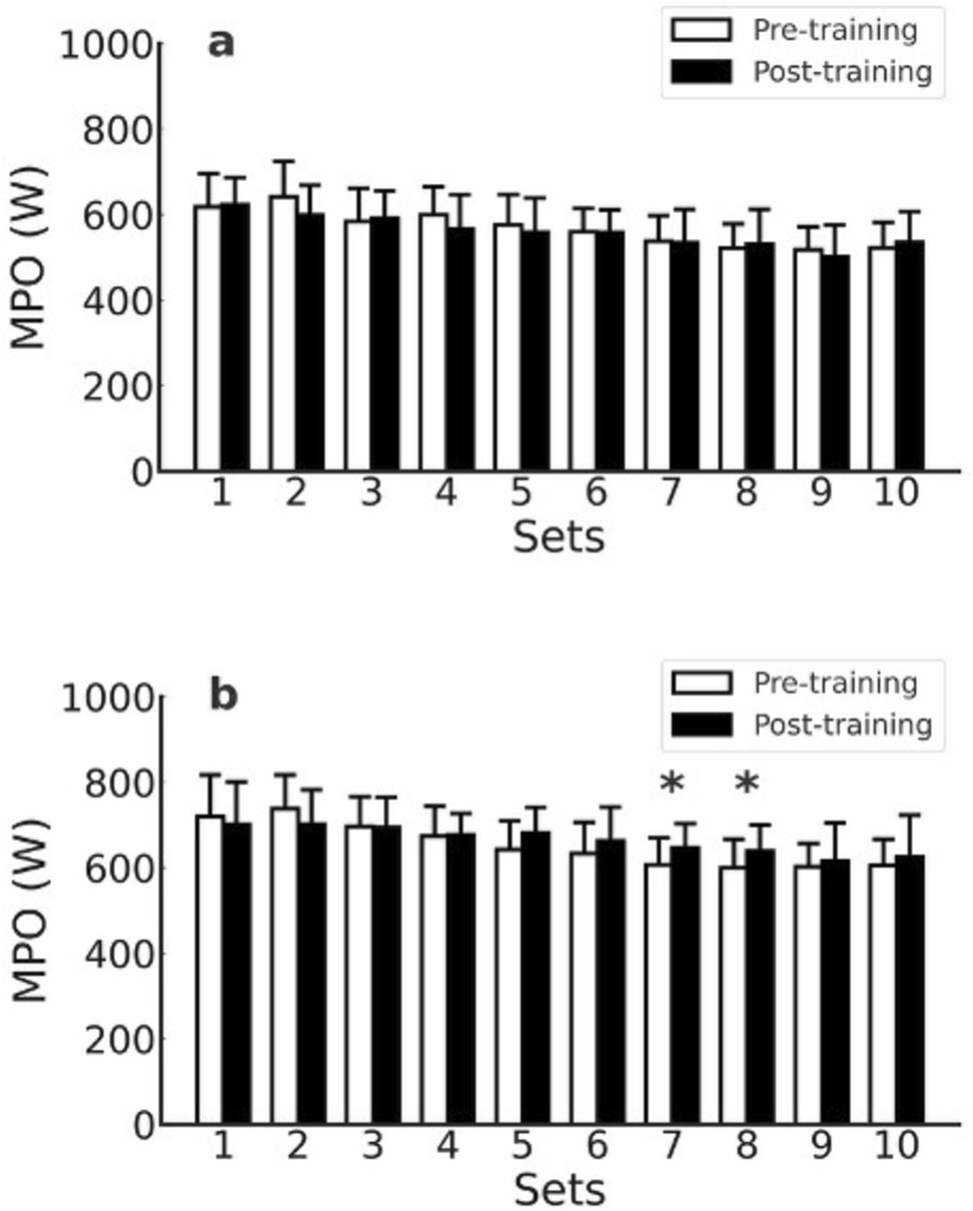
Mean Power Output (MPO) of CON group **a** and VHL group **b** in the RSA test Values are means ± SD * Significant difference between Pre-training and Post-training (n = 7 per group)

Peak power output (PPO) and mean power output (MPO) were assessed as indicators of RSA performance. PPO did not differ between groups at baseline and showed no significant Pre–Post changes in either group. In contrast, within the VHL group (Fig. 1b) MPO was higher Post-training than Pre-training, particularly during the later repetitions of the 10 sprints. Significant within-group improvements were observed in the 7th and 8th sprints compared with Pre-training values (asterisks indicate significant differences). No significant Pre–Post changes in MPO were observed in the CON group (Fig. 1a).

### Aerobic capacity (VO₂max and oxygen pulse)

Table 1 summarizes the changes in VO₂max Pre-training and Post-training. VO₂max significantly increased from Pre- to Post-training within the VHL group (p < 0.01), whereas no significant change was observed within the CON group. A significant group × time interaction was also found (p < 0.05, η^2^ = 0.27), indicating that VO₂max increased to a greater extent in the VHL group than in the CON group. Independent-samples t-tests confirmed no significant baseline differences in VO₂max, MAOD, or RSA between the CON and VHL groups (all p > 0.05).

**Table 1:** Change in maximal oxygen uptake (VO₂max) and maximal accumulated oxygen deficit (MAOD) Pre-training and Post-training in the CON and VHL groups

	CON group	VHL group
VO₂max (ml/min)		
Pre-training	2494.8 ± 271.1	2857.3 ± 395.3
Post-training	2605.1 ± 179.1	3141.3 ± 403.9*
MAOD (ml/min/kg)		
Pre-training	772.5 ± 133.6	735.0 ± 116.2
Post-training	793.0 ± 132.4	735.5 ± 142.3

Values are means ± SD * significantly different from Pre-training

Independent-samples t-tests revealed no significant baseline differences between groups (all p > 0.05)

Table 2 presents VO₂ and oxygen pulse during the RSA test. Within the VHL group, oxygen pulse significantly increased from Pre- to Post-training. Concomitantly, VO₂ during the RSA test also increased (p < 0.05), indicating that the rise in oxygen pulse reflected enhanced VO₂. No significant changes were observed in the CON group.

**Table 2:** Heart rate, oxygen uptake (VO₂), and oxygen pulse during the RSA test Pre-training and Post-training in the CON and VHL groups

	CON group		VHL group	
	Pre-training	Post-training	Pre-training	Post-training
HR (bpm)	151.2 ± 7.1	152.2 ± 4.4	158.9 ± 3.7	159.0 ± 4.5
VO₂(ml/min)	1765.9 ± 245.1	1901.4 ± 166.7	1882.2 ± 241.2	2224.7 ± 256.9^*^
O2 pulse (ml/beat)	12.8 ± 0.7	12.8 ± 0.5	12.6 ± 1.6	14.1 ± 1.4^*^

Values are means ± SD * significantly different from Pre-training.

**Fig. 2:**
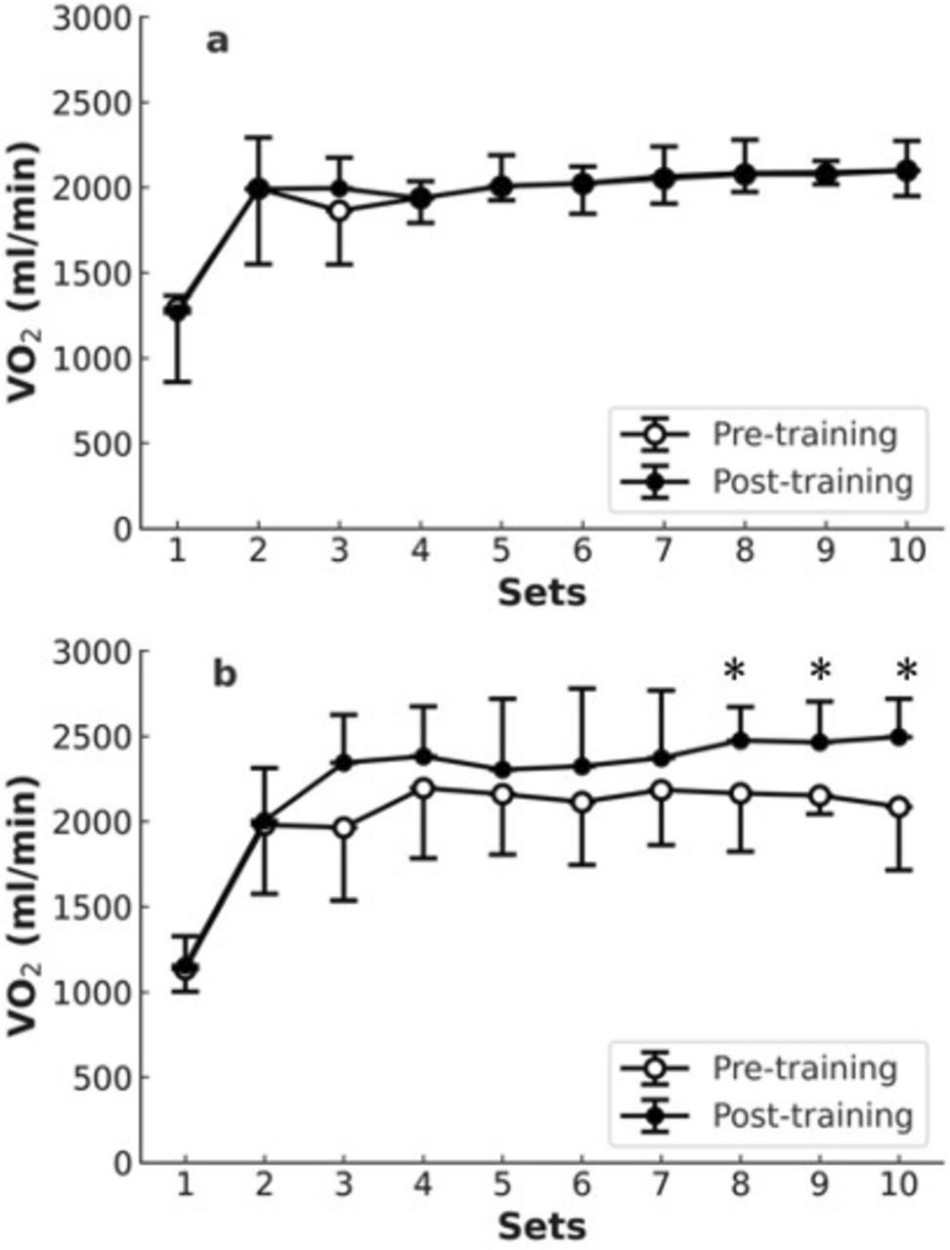
Change in oxygen uptake (VO₂) of CON group **a** and VHL group **b** in the RSA test Values are means ± SD * Significantly different from Pre-training (n = 7 per group)

Figure 2 shows VO₂ during each repetition of the RSA test. In the VHL group, VO₂ values were higher Post-training compared with Pre-training, particularly from the third repetition onward. Significant increases were observed in the 8th, 9th, and 10th sprints (p < 0.05).

#### Anaerobic capacity (MAOD)

As shown in Table 1, MAOD did not change significantly in either group. These findings indicate that the improvements in mean power output during the RSA test in the VHL group were likely due to enhanced aerobic capacity and associated improvements in energy supply efficiency, rather than to increases in anaerobic capacity.

## Discussion

The present study investigated the effects of sprint interval training combined with voluntary hypoventilation (VHL) on aerobic and anaerobic energy metabolism. The main findings were that VO₂max and oxygen pulse significantly increased, and repeated sprint ability (RSA; mean power output) improved in the VHL group after three weeks of training. These findings indicate that VHL provides a practical and effective training stimulus to enhance aerobic energy metabolism, even within a relatively short training period.

### Cardiopulmonary and peripheral adaptations

The observed increases in VO₂max and oxygen pulse in the VHL group suggest improvements in cardiopulmonary function and peripheral oxygen utilization. VHL induces a transient hypoxemic state through ventilatory restriction, which may enhance compensatory cardiovascular responses such as increased cardiac output and capillary perfusion (Woorons et al. 2010). These responses resemble those reported in altitude training (654 Wüthrich et al. 2015). Improvements in VO₂max are generally attributed to increased cardiac output and a widened arteriovenous oxygen difference (a-vO₂ diff) (Bassett and Howley 2000; Saltin and Calbet 2006; Lundby et al. 2017). In the present study, oxygen pulse significantly increased, supporting the interpretation that stroke volume increased. Furthermore, given the short 3-week training period, rapid adaptations, such as increases in mitochondrial content, may have contributed to enhanced peripheral oxygen utilization, as previously reported in similar high-intensity interval training studies. Indeed, Mølmen et al. (2025) reported that similar high-intensity interval training rapidly increased mitochondrial abundance in skeletal muscle. In addition, endurance training under hypoxic conditions has been shown to induce both angiogenesis and mitochondrial biogenesis (Vogt et al. 2001), which is consistent with the present findings.

### Repeated sprint ability and MAOD

The present study found improvements in mean power output during the RSA test in the VHL group. Although RSA involves short-duration, high-intensity exercise, MAOD remained unchanged in both groups. Thus, the improvement in mean power output is unlikely to reflect enhanced anaerobic metabolic capacity. This lack of change in MAOD is likely attributable to the relatively short training duration. Instead, the improvement in RSA performance is more plausibly explained by enhanced aerobic capacity (VO₂max), which may have facilitated more efficient energy resynthesis between sprints (e.g., faster phosphocreatine recovery) and increased fatigue tolerance (e.g., greater lactate oxidation). This interpretation is supported by the finding that VO₂ during the RSA test was significantly higher in the VHL group Post-training, particularly during the later sprints. These adaptations may contribute to sustaining power output during the final phases of competition and to promoting recovery between efforts.

### Hypoxemia and molecular adaptations

The decrease in SpO₂ observed during VHL training indicates that a mild hypoxemic state was induced. This transient hypoxemia likely resulted from ventilatory restriction and imposed hypoxic metabolic stress. Such transient hypoxia has been shown to promote molecular adaptations, including the activation of hypoxia-inducible factor-1α (HIF-1α) (Faiss et al. 2013). Moreover, Ferraro et al. (2014) reported that transient hypoxia during exercise increases the production of reactive oxygen species (ROS), which stabilizes HIF-1α and, via AMPK and Ca^2^⁺ signaling pathways, induces PGC-1α expression, thereby facilitating angiogenesis and mitochondrial biogenesis. This proposed mechanism is consistent with the present finding that VHL training enhanced aerobic metabolic capacity.

Although both VHL and altitude or artificial hypoxic training impose a reduction in arterial oxygen availability, the physiological mechanisms underlying these responses differ. VHL induces a rapid and transient desaturation through voluntary ventilatory restriction, whereas altitude and hypoxic chamber exposure elicit a more sustained reduction in inspired PO₂. Despite these differences, both strategies appear to activate similar downstream pathways related to oxygen transport and metabolic regulation, including HIF-1α stabilization, VEGF-mediated angiogenesis, and PGC-1α–related mitochondrial adaptations, as documented in hypoxic sprint studies (Faiss et al. 2013; Girard et al. 2017). Notably, VHL may produce a sharper but shorter hypoxemic episode during each sprint, potentially leading to repeated bouts of metabolic stress comparable to the intermittent hypoxic stimulus seen in RSH protocols. Given that VHL can induce these hypoxia-related adaptations without requiring specialized equipment or environmental modification, it may represent a practical alternative to hypoxic training modalities, particularly in laboratory or field settings.

### Lack of adaptations in the CON group

In contrast, no significant changes were observed in either aerobic or anaerobic indices in the CON group. This may be explained by the fact that sprint training with normal breathing does not induce sufficient hypoxic stimulation or dyspnea, thereby imposing relatively lower metabolic and circulatory stress (Woorons et al. 2010). Faiss et al. (2013) similarly reported that repeated sprint training under normoxic conditions induces only limited performance improvements, whereas hypoxic conditions elicit more pronounced effects. Consistently, Girard et al. (2017) concluded in their review that repeated sprint training in hypoxia effectively induces metabolic adaptations and improves performance, whereas training in normoxia results in limited benefits. Trincat et al. (2017) also emphasized that voluntary hypoventilation is necessary to elicit such adaptations, and that sprint training without respiratory restriction may be insufficient.

From a physiological perspective, sprint exercise under normal breathing appears to provide insufficient hypoxic stimulation to fully activate molecular pathways such as HIF-1α and VEGF, which are essential for angiogenesis and oxygen transport. Furthermore, molecular adaptations such as PGC-1α–mediated mitochondrial biogenesis and the upregulation of oxidative enzymes have been reported to occur more robustly under hypoxic conditions (Faiss et al. 2013; Zoll et al. 2006; Hoppeler & Vogt 2001; Lundby et al. 2006; Vogt et al. 2001). Therefore, the absence of significant changes in the CON group is likely attributable to insufficient hypoxic stress to exceed the adaptive threshold during the relatively short intervention period.

## Limitations

Several limitations of the present study should be noted. First, the sample size was small, with seven participants in each group, which may have reduced statistical power. However, this sample size is comparable to previous VHL and hypoventilation-based training studies (typically n = 6–10), and the present study should therefore be regarded as pilot evidence that can guide larger trials. Second, the intervention period was relatively short (three weeks; six sessions), so long-term adaptations could not be fully assessed. This duration was chosen based on earlier VHL and repeated-sprint hypoxia work showing significant adaptations after a similar number of sessions (e.g., Faiss et al. 2013; Trincat et al. 2017; Woorons et al. 2019). Nevertheless, longer interventions may reveal additional adaptations, such as changes in anaerobic enzyme activity or muscle morphology, and future studies should therefore employ longer training periods to examine sustained effects.

Third, although MAOD was measured as an index of anaerobic capacity, blood lactate concentration and skeletal muscle biopsies for metabolic enzyme activity were not assessed. Consequently, interpretations of anaerobic adaptations remain limited. Future studies that incorporate biochemical and molecular analyses (e.g., expression of PGC-1α and HIF-1α in skeletal muscle) would provide further insight into the mechanisms underlying VHL-induced adaptations. Future studies combining VHL interventions with muscle biopsies and molecular analyses (e.g., HIF-1α, PGC-1α, and key aerobic and anaerobic enzymes) are needed to clarify the mechanisms underlying the observed adaptations.

Therefore, the findings of this study should be considered preliminary evidence based on a small sample size and a short-term intervention. Nevertheless, the demonstration that a simple VHL training protocol can improve aerobic capacity and RSA highlights the importance of conducting larger-scale and longer-term investigations. Finally, because our participants were healthy young men, the generalizability of these findings to women, older adults, or clinical populations remains uncertain and should be tested in future studies.

## Conclusion

The present study provides evidence that sprint interval training combined with voluntary hypoventilation (VHL) improves aerobic capacity (VO₂max and oxygen pulse) and repeated sprint ability (mean power output) in young men. In the VHL group, VO₂ during recovery and during the latter repetitions of the RSA test significantly increased, suggesting improved metabolic responses and enhanced fatigue tolerance. In contrast, no significant changes were observed in maximal accumulated oxygen deficit (MAOD), suggesting that the short intervention period was insufficient to induce improvements in anaerobic capacity.

These findings indicate that VHL elicits adaptations similar to those achieved through altitude training or artificial hypoxic environments, while offering the practical advantage of requiring neither relocation nor specialized equipment. Moreover, because VHL can be safely implemented through voluntary control of breathing, it may be applicable not only for enhancing athletic performance but also, as suggested by previous studies, for promoting health benefits such as improved lipid metabolism and obesity prevention. Collectively, VHL sprint training may serve as an effective strategy for both competitive athletes and the general population.

## Data Availability

The datasets generated during the current study are not publicly available due to ethical restrictions related to participant confidentiality but are available from the corresponding author on reasonable request.

## Abbreviations

CON: Control (normal breathing group)
MAOD: Maximal accumulated oxygen deficit
MPO: Mean power output
PPO: Peak power output
RSA: Repeated sprint ability
SpO₂: Arterial oxygen saturation
VHL: Voluntary hypoventilation in low lung volume
VO₂max: Maximal oxygen uptake
